# Supplementary material for: Coevolution of Rumen Epithelial circRNAs with Their Microbiota and Metabolites in Response to Cold-Season Nutritional Stress in Tibetan Sheep
Source: Int J Mol Sci. 2022 Sep 10;23(18):10488. doi: 10.3390/ijms231810488 (PMC9499677; doi:10.3390/ijms231810488)
Supplement: Supplementary file 1 [file ijms-23-10488-s001.zip › ijms-1879133-supplementary.pdf]

Table S1. Data quality control statistics.

Samples	Read Number	Base Number	GC Content	%≥Q30
cold1	53,859,439	16,025,456,748	48.37%	98.27%
cold2	60,990,364	18,103,396,314	48.85%	98.34%
cold3	61,090,369	18,095,280,638	48.28%	98.22%
warm1	52,304,300	15,558,868,816	50.68%	98.37%
warm2	54,632,262	16,233,111,642	51.21%	98.38%
warm3	58,382,319	17,397,315,876	50.83%	98.42%

Table S2. Reference genome alignment read statistics.

Samples	Total Reads	Mapped Reads	Uniq Reads	Multiple Map Reads
cold1	107,718,878	106,466,546(98.84%)	66,365,958(61.61%)	40,100,588(37.23%)
cold2	121,980,728	120,562,798(98.84%)	75,424,091(61.83%)	45,138,707(37.00%)
cold3	122,180,738	120,615,112(98.72%)	76,646,321(62.73%)	43,968,791(35.99%)
warm1	104,608,600	100,657,298(96.22%)	48,032,801(45.92%)	52,624,497(50.31%)
warm2	109,264,524	103,853,500(95.05%)	48,383,138(44.28%)	55,470,362(50.77%)
warm3	116,764,638	113,366,154(97.09%)	61,128,334(52.35%)	52,237,820(44.74%)

Table S3. Expression pattern of differential circRNA in rumen epithelium of Tibetan sheep during cold and warm seasons.

#ID	cold1_TPM	cold2_TPM	cold3_TPM	warm1_TPM	warm2_TPM	warm3_TPM	P-value	log2FC	Regulated(In cold season)
NC_040252.1:36597508|36635581	0.323081	0.739964	0.401743	0.204194	0	0	0.002795	-3.0361	up
NC_040252.1:45254209|45255940	0	0	0	0.544516	0.666594	0.661829	0.000935	4.899293	down
NC_040252.1:72173631|72216643	0.129233	0.170761	0.344351	0	0	0	0.009989	-4.09456	up
NC_040252.1:73942400|73951504	0.710779	0.455363	0.688703	0	0	0.300831	0.000943	-2.94905	up
NC_040252.1:99688247|99688502	0.452314	0.512283	1.20523	0	0.266638	0.360998	0.007697	-2.19415	up
NC_040252.1:132512083|132514812	0	0	0.114784	0.680645	0.666594	0.721995	0.002795	3.196198	down
NC_040253.1:31909981|31938690	0.193849	0.341522	0.114784	0	0	0	0.009905	-4.09815	up
NC_040253.1:45701039|45703283	0.258465	0.227681	0.344351	0	0	0	0.003469	-4.50502	up
NC_040253.1:95968449|95969159	2.132337	2.049132	1.664365	1.089033	1.199869	1.383824	0.002244	-1.17289	up
NC_040253.1:96378897|96382566	0	0	0	0.204194	0.599934	0.240665	0.00922	4.095717	down
NC_040253.1:102980470|103003675	2.649267	3.984424	1.893933	2.382259	1.666485	1.804988	0.007387	-1.03371	up
NC_040253.1:161848004|161855481	0.646163	0.853805	0.975662	0.476452	0.266638	0.300831	0.004586	-1.71324	up
NC_040254.1:13141709|13192404	0	0	0.114784	0.476452	0.666594	0.96266	0.002989	3.187698	down
NC_040254.1:13288207|13289786	1.938488	2.162973	1.49219	0	0	0	5.51E-08	-7.07562	up
NC_040254.1:75223248|75230903	0.193849	0.170761	0.172176	1.020968	0.866572	1.263491	0.004345	1.944482	down

#ID	cold1_TPM	cold2_TPM	cold3_TPM	warm1_TPM	warm2_TPM	warm3_TPM	P-value	log2FC	Regulated(In cold season)
NC_040252.1:36597508|36635581	0.323081	0.739964	0.401743	0.204194	0	0	0.002795	-3.0361	up
NC_040252.1:45254209|45255940	0	0	0	0.544516	0.666594	0.661829	0.000935	4.899293	down
NC_040252.1:72173631|72216643	0.129233	0.170761	0.344351	0	0	0	0.009989	-4.09456	up
NC_040252.1:73942400|73951504	0.710779	0.455363	0.688703	0	0	0.300831	0.000943	-2.94905	up
NC_040252.1:99688247|99688502	0.452314	0.512283	1.20523	0	0.266638	0.360998	0.007697	-2.19415	up
NC_040252.1:132512083|132514812	0	0	0.114784	0.680645	0.666594	0.721995	0.002795	3.196198	down
NC_040253.1:31909981|31938690	0.193849	0.341522	0.114784	0	0	0	0.009905	-4.09815	up
NC_040253.1:45701039|45703283	0.258465	0.227681	0.344351	0	0	0	0.003469	-4.50502	up
NC_040253.1:95968449|95969159	2.132337	2.049132	1.664365	1.089033	1.199869	1.383824	0.002244	-1.17289	up
NC_040253.1:96378897|96382566	0	0	0	0.204194	0.599934	0.240665	0.00922	4.095717	down
NC_040253.1:102980470|103003675	2.649267	3.984424	1.893933	2.382259	1.666485	1.804988	0.007387	-1.03371	up
NC_040253.1:161848004|161855481	0.646163	0.853805	0.975662	0.476452	0.266638	0.300831	0.004586	-1.71324	up
NC_040254.1:112514514|112521478	0.193849	0.341522	0.229568	0	0	0	0.004979	-4.37269	up
NC_040254.1:181581936|181590859	0.51693	0.683044	0.229568	1.905807	1.799803	2.767648	0.000718	1.615617	down
NC_040254.1:209964956|209973020	1.680023	1.309168	1.893933	0.544516	0.666594	1.804988	0.005082	-1.24008	up
NC_040255.1:29810624|29825377	0.646163	0.569203	1.320014	3.60742	2.999672	2.466817	0.004852	1.323972	down
NC_040255.1:70391806|70408315	0.129233	0.227681	0	0.680645	0.866572	1.143159	0.005263	2.246571	down
NC_040255.1:114785769|114803933	1.292326	0.739964	0.401743	0	0	0	1.61E-05	-5.99003	up

#ID	cold1_TPM	cold2_TPM	cold3_TPM	warm1_TPM	warm2_TPM	warm3_TPM	P-value	log2FC	Regulated(In cold season)
NC_040252.1:36597508|36635581	0.323081	0.739964	0.401743	0.204194	0	0	0.002795	-3.0361	up
NC_040252.1:45254209|45255940	0	0	0	0.544516	0.666594	0.661829	0.000935	4.899293	down
NC_040252.1:72173631|72216643	0.129233	0.170761	0.344351	0	0	0	0.009989	-4.09456	up
NC_040252.1:73942400|73951504	0.710779	0.455363	0.688703	0	0	0.300831	0.000943	-2.94905	up
NC_040252.1:99688247|99688502	0.452314	0.512283	1.20523	0	0.266638	0.360998	0.007697	-2.19415	up
NC_040252.1:132512083|132514812	0	0	0.114784	0.680645	0.666594	0.721995	0.002795	3.196198	down
NC_040253.1:31909981|31938690	0.193849	0.341522	0.114784	0	0	0	0.009905	-4.09815	up
NC_040253.1:45701039|45703283	0.258465	0.227681	0.344351	0	0	0	0.003469	-4.50502	up
NC_040253.1:95968449|95969159	2.132337	2.049132	1.664365	1.089033	1.199869	1.383824	0.002244	-1.17289	up
NC_040253.1:96378897|96382566	0	0	0	0.204194	0.599934	0.240665	0.00922	4.095717	down
NC_040253.1:102980470|103003675	2.649267	3.984424	1.893933	2.382259	1.666485	1.804988	0.007387	-1.03371	up
NC_040253.1:161848004|161855481	0.646163	0.853805	0.975662	0.476452	0.266638	0.300831	0.004586	-1.71324	up
NC_040255.1:124730726|124751843	0.258465	0.284602	0.573919	0	0	0	0.000909	-4.9486	up
NC_040256.1:6951961|6954445	0	0	0	0.340323	0.399956	0.360998	0.008056	4.146662	down
NC_040256.1:74599651|74601345	0.193849	0.284602	0.172176	0	0	0	0.00947	-4.11532	up
NC_040256.1:78451819|78454934	0.258465	0.398442	0.114784	0	0	0	0.005142	-4.36314	up
NC_040257.1:61222433|61223481	0	0	0	0.816774	0.199978	0.300831	0.004471	4.381501	down
NC_040257.1:64017417|64022902	0	0	0	0.204194	0.266638	0.661829	0.009716	4.072788	down

#ID	cold1_TPM	cold2_TPM	cold3_TPM	warm1_TPM	warm2_TPM	warm3_TPM	P-value	log2FC	Regulated(In cold season)
NC_040252.1:36597508|36635581	0.323081	0.739964	0.401743	0.204194	0	0	0.002795	-3.0361	up
NC_040252.1:45254209|45255940	0	0	0	0.544516	0.666594	0.661829	0.000935	4.899293	down
NC_040252.1:72173631|72216643	0.129233	0.170761	0.344351	0	0	0	0.009989	-4.09456	up
NC_040252.1:73942400|73951504	0.710779	0.455363	0.688703	0	0	0.300831	0.000943	-2.94905	up
NC_040252.1:99688247|99688502	0.452314	0.512283	1.20523	0	0.266638	0.360998	0.007697	-2.19415	up
NC_040252.1:132512083|132514812	0	0	0.114784	0.680645	0.666594	0.721995	0.002795	3.196198	down
NC_040253.1:31909981|31938690	0.193849	0.341522	0.114784	0	0	0	0.009905	-4.09815	up
NC_040253.1:45701039|45703283	0.258465	0.227681	0.344351	0	0	0	0.003469	-4.50502	up
NC_040253.1:95968449|95969159	2.132337	2.049132	1.664365	1.089033	1.199869	1.383824	0.002244	-1.17289	up
NC_040253.1:96378897|96382566	0	0	0	0.204194	0.599934	0.240665	0.00922	4.095717	down
NC_040253.1:102980470|103003675	2.649267	3.984424	1.893933	2.382259	1.666485	1.804988	0.007387	-1.03371	up
NC_040253.1:161848004|161855481	0.646163	0.853805	0.975662	0.476452	0.266638	0.300831	0.004586	-1.71324	up
NC_040258.1:353138|353385	0.323081	0.170761	0.229568	0	0	0	0.006364	-4.27741	up
NC_040258.1:11318705|11337918	0.452314	0.512283	0.401743	1.565484	0.999891	3.970973	0.002456	1.614978	down
NC_040258.1:35255740|35256250	0	0	0	1.089033	0.333297	0.120333	0.003414	4.512115	down
NC_040259.1:83405933|83406542	0.775395	0.284602	0.573919	1.020968	2.266419	3.128645	0.009168	1.404791	down
NC_040259.1:93584922|93650681	0	0	0.114784	0.544516	0.666594	0.721995	0.004409	3.092543	down

#ID	cold1_TPM	cold2_TPM	cold3_TPM	warm1_TPM	warm2_TPM	warm3_TPM	P-value	log2FC	Regulated(In cold season)
NC_040252.1:36597508|36635581	0.323081	0.739964	0.401743	0.204194	0	0	0.002795	-3.0361	up
NC_040252.1:45254209|45255940	0	0	0	0.544516	0.666594	0.661829	0.000935	4.899293	down
NC_040252.1:72173631|72216643	0.129233	0.170761	0.344351	0	0	0	0.009989	-4.09456	up
NC_040252.1:73942400|73951504	0.710779	0.455363	0.688703	0	0	0.300831	0.000943	-2.94905	up
NC_040252.1:99688247|99688502	0.452314	0.512283	1.20523	0	0.266638	0.360998	0.007697	-2.19415	up
NC_040252.1:132512083|132514812	0	0	0.114784	0.680645	0.666594	0.721995	0.002795	3.196198	down
NC_040253.1:31909981|31938690	0.193849	0.341522	0.114784	0	0	0	0.009905	-4.09815	up
NC_040253.1:45701039|45703283	0.258465	0.227681	0.344351	0	0	0	0.003469	-4.50502	up
NC_040253.1:95968449|95969159	2.132337	2.049132	1.664365	1.089033	1.199869	1.383824	0.002244	-1.17289	up
NC_040253.1:96378897|96382566	0	0	0	0.204194	0.599934	0.240665	0.00922	4.095717	down
NC_040253.1:102980470|103003675	2.649267	3.984424	1.893933	2.382259	1.666485	1.804988	0.007387	-1.03371	up
NC_040253.1:161848004|161855481	0.646163	0.853805	0.975662	0.476452	0.266638	0.300831	0.004586	-1.71324	up
NC_040262.1:21285158|21292466	0	0.227681	0	8.848389	9.532292	6.798787	2.41E-13	5.881712	down
NC_040262.1:25898501|25900203	1.356942	0.569203	1.033054	0	0	0	4.51E-06	-6.25843	up
NC_040262.1:53244324|53251250	0.646163	0.569203	0.573919	2.110001	1.933122	2.165985	0.004523	1.264301	down
NC_040264.1:34000774|34006459	0	0	0.114784	0.544516	0.666594	0.601663	0.006181	3.012258	down
NC_040266.1:22326033|22348567	0	0	0	0.272258	0.333297	0.661829	0.00584	4.275468	down

#ID	cold1_TPM	cold2_TPM	cold3_TPM	warm1_TPM	warm2_TPM	warm3_TPM	P-value	log2FC	Regulated(In cold season)
NC_040252.1:36597508|36635581	0.323081	0.739964	0.401743	0.204194	0	0	0.002795	-3.0361	up
NC_040252.1:45254209|45255940	0	0	0	0.544516	0.666594	0.661829	0.000935	4.899293	down
NC_040252.1:72173631|72216643	0.129233	0.170761	0.344351	0	0	0	0.009989	-4.09456	up
NC_040252.1:73942400|73951504	0.710779	0.455363	0.688703	0	0	0.300831	0.000943	-2.94905	up
NC_040252.1:99688247|99688502	0.452314	0.512283	1.20523	0	0.266638	0.360998	0.007697	-2.19415	up
NC_040252.1:132512083|132514812	0	0	0.114784	0.680645	0.666594	0.721995	0.002795	3.196198	down
NC_040253.1:31909981|31938690	0.193849	0.341522	0.114784	0	0	0	0.009905	-4.09815	up
NC_040253.1:45701039|45703283	0.258465	0.227681	0.344351	0	0	0	0.003469	-4.50502	up
NC_040253.1:95968449|95969159	2.132337	2.049132	1.664365	1.089033	1.199869	1.383824	0.002244	-1.17289	up
NC_040253.1:96378897|96382566	0	0	0	0.204194	0.599934	0.240665	0.00922	4.095717	down
NC_040253.1:102980470|103003675	2.649267	3.984424	1.893933	2.382259	1.666485	1.804988	0.007387	-1.03371	up
NC_040253.1:161848004|161855481	0.646163	0.853805	0.975662	0.476452	0.266638	0.300831	0.004586	-1.71324	up
NC_040268.1:2305136|2332134	1.421558	1.479929	1.20523	0.340323	0.599934	0.842328	0.000399	-1.69904	up
NC_040269.1:40762104|40775219	0	0	0	0.476452	0.666594	1.143159	0.000455	5.114934	down
NC_040269.1:68309959|68364662	0.387698	0.398442	0.401743	0	0	0	0.000646	-5.04661	up
NC_040269.1:70665914|70676391	2.455418	7.798087	0.746095	0	0.266638	0	0.001513	-4.41655	up
NC_040271.1:12416268|12417457	0.258465	0.170761	0.114784	0.544516	0.733253	1.92532	0.008264	1.895479	down
NC_040271.1:29667395|29688230	0.193849	0.170761	0.573919	0	0	0	0.002191	-4.671	up

#ID	cold1_TPM	cold2_TPM	cold3_TPM	warm1_TPM	warm2_TPM	warm3_TPM	P-value	log2FC	Regulated(In cold season)
NC_040252.1:36597508|36635581	0.323081	0.739964	0.401743	0.204194	0	0	0.002795	-3.0361	up
NC_040252.1:45254209|45255940	0	0	0	0.544516	0.666594	0.661829	0.000935	4.899293	down
NC_040252.1:72173631|72216643	0.129233	0.170761	0.344351	0	0	0	0.009989	-4.09456	up
NC_040252.1:73942400|73951504	0.710779	0.455363	0.688703	0	0	0.300831	0.000943	-2.94905	up
NC_040252.1:99688247|99688502	0.452314	0.512283	1.20523	0	0.266638	0.360998	0.007697	-2.19415	up
NC_040252.1:132512083|132514812	0	0	0.114784	0.680645	0.666594	0.721995	0.002795	3.196198	down
NC_040253.1:31909981|31938690	0.193849	0.341522	0.114784	0	0	0	0.009905	-4.09815	up
NC_040253.1:45701039|45703283	0.258465	0.227681	0.344351	0	0	0	0.003469	-4.50502	up
NC_040253.1:95968449|95969159	2.132337	2.049132	1.664365	1.089033	1.199869	1.383824	0.002244	-1.17289	up
NC_040253.1:96378897|96382566	0	0	0	0.204194	0.599934	0.240665	0.00922	4.095717	down
NC_040253.1:102980470|103003675	2.649267	3.984424	1.893933	2.382259	1.666485	1.804988	0.007387	-1.03371	up
NC_040253.1:161848004|161855481	0.646163	0.853805	0.975662	0.476452	0.266638	0.300831	0.004586	-1.71324	up
NC_040271.1:51577985|51588160	0	0	0	0.204194	0.399956	0.721995	0.005071	4.331114	down
NC_040272.1:42750220|42750603	0.387698	0.227681	0.631311	0	0	0.120333	0.002939	-3.38763	up
NC_040272.1:45473605|45473760	0.840012	0.398442	0.631311	0.272258	0	0	0.006894	-2.89961	up
NC_040272.1:47422771|47448326	0	0	0	0.476452	0.333297	0.661829	0.002989	4.519516	down
NC_040274.1:49975759|49979215	1.809256	2.789097	2.066109	1.089033	1.399847	1.143159	0.000477	-1.36501	up
NC_040275.1:28680890|28683112	1.098477	1.195327	1.033054	0.136129	0	0.360998	3.22E-06	-3.14879	up
NC_040276.1:10040393|10059385	0	0	0	0.204194	0.666594	0.661829	0.002493	4.58717	down

#ID	cold1_TPM	cold2_TPM	cold3_TPM	warm1_TPM	warm2_TPM	warm3_TPM	P-value	log2FC	Regulated(In cold season)
NC_040252.1:36597508|36635581	0.323081	0.739964	0.401743	0.204194	0	0	0.002795	-3.0361	up
NC_040252.1:45254209|45255940	0	0	0	0.544516	0.666594	0.661829	0.000935	4.899293	down
NC_040252.1:72173631|72216643	0.129233	0.170761	0.344351	0	0	0	0.009989	-4.09456	up
NC_040252.1:73942400|73951504	0.710779	0.455363	0.688703	0	0	0.300831	0.000943	-2.94905	up
NC_040252.1:99688247|99688502	0.452314	0.512283	1.20523	0	0.266638	0.360998	0.007697	-2.19415	up
NC_040252.1:132512083|132514812	0	0	0.114784	0.680645	0.666594	0.721995	0.002795	3.196198	down
NC_040253.1:31909981|31938690	0.193849	0.341522	0.114784	0	0	0	0.009905	-4.09815	up
NC_040253.1:45701039|45703283	0.258465	0.227681	0.344351	0	0	0	0.003469	-4.50502	up
NC_040253.1:95968449|95969159	2.132337	2.049132	1.664365	1.089033	1.199869	1.383824	0.002244	-1.17289	up
NC_040253.1:96378897|96382566	0	0	0	0.204194	0.599934	0.240665	0.00922	4.095717	down
NC_040253.1:102980470|103003675	2.649267	3.984424	1.893933	2.382259	1.666485	1.804988	0.007387	-1.03371	up
NC_040253.1:161848004|161855481	0.646163	0.853805	0.975662	0.476452	0.266638	0.300831	0.004586	-1.71324	up
								3	
NC_040278.1:17366023|17388922	0.193849	0.113841	0	0.612581	0.533275	1.323658	0.007936	2.29698	down
NC_040278.1:73454389|73479763	1.873872	1.536849	2.640028	1.157097	1.199869	1.744821	0.00522	-1.08004	up
NC_040278.1:75841464|75860924	0.710779	0.284602	0.573919	1.157097	2.066441	3.429477	0.0024	1.5072	down
NC_040278.1:76109122|76109357	0.387698	0.398442	0.688703	0.136129	0	0.180499	0.005042	-2.56257	up
NC_040278.1:103131586|103133048	7.10779	2.447575	11.42099	1.905807	2.866354	0.842328	0.005609	-2.18798	up
NC_040278.1:136801496|136806231	0	0	0	0.340323	0.266638	1.082993	0.002247	4.624177	down
